# Peripheral blood-based cell signature indicates response to interstitial brachytherapy in primary liver cancer

**DOI:** 10.1007/s00432-023-04875-z

**Published:** 2023-05-29

**Authors:** Sophia Kästle, Matthias R. Stechele, Lisa Richter, Regina Schinner, Elif Öcal, Marianna Alunni-Fabbroni, Enrico De Toni, Stefanie Corradini, Max Seidensticker, S. Nahum Goldberg, Jens Ricke, Moritz Wildgruber, Melanie A. Kimm

**Affiliations:** 1 Department of Radiology, University Hospital, LMU Munich, Munich, Germany; 2 Core Facility Flow Cytometry, Biomedical Center Munich, Ludwig-Maximilians-Universität München, Planegg-Martinsried, Germany; 3 Department of Medicine II, University Hospital, LMU Munich, Munich, Germany; 4 Department of Radiation Oncology, University Hospital, LMU Munich, Munich, Germany; 5 Goldyne Savad Institute of Gene Therapy, Hadassah Hebrew University Hospital, Jerusalem, Israel; 6 Laboratory for Minimally Invasive Tumor Therapies, Department of Radiology, Beth Israel Deaconess Medical Center, Harvard Medical School, Boston, MA USA; 7 Division of Image-Guided Therapy and Interventional Oncology, Department of Radiology, Hadassah Hebrew University Hospital, Jerusalem, Israel

**Keywords:** Liver cancer, Brachytherapy, Blood cells, Biomarker, Flow cytometry, Response prediction

## Abstract

**Purpose:**

Biomarkers are essential to implement personalized therapies in cancer treatment options. As primary liver tumors are increasing and treatment is coupled to liver function and activation of systemic cells of the immune system, we investigated blood-based cells for their ability to predict response to local ablative therapy.

**Methods:**

We analyzed peripheral blood cells in 20 patients with primary liver cancer at baseline and following brachytherapy. In addition to platelets, leukocytes, lymphocytes, monocytes, neutrophils and most common ratios PLR, LMR, NMR and NLR, we investigated T cell and NKT cell populations of 11 responders and 9 non-responders using flow cytometry.

**Results:**

We have found a peripheral blood cell signature that differed significantly between responders and non-responders treated with interstitial brachytherapy (IBT). At baseline, non-responders featured higher numbers of platelets, monocytes and neutrophils, a higher platelet-to-lymphocyte ratio and an increase in the NKT cell population with a concurrent reduction in CD16 + NKT cells. Simultaneously, a lower percentage of CD4 + T cells was present in non-responders, as also reflected in a lower CD4/8 ratio. CD45RO + memory cells were lower in both, CD4 + and CD8 + T cell populations whereas PD-1 + T cells were only present in the CD4 + T cell population.

**Conclusion:**

Baseline blood-based cell signature may function as a biomarker to predict response following brachytherapy in primary liver cancer.

**Supplementary Information:**

The online version contains supplementary material available at 10.1007/s00432-023-04875-z.

## Introduction

Currently, liver cancer is among the five most common causes of cancer death worldwide and the incidence is rising (Rumgay et al. 2022). Primary liver cancers mainly comprise hepatocellular carcinoma (HCC) with about 75–85% and intrahepatic cholangiocarcinoma (iCCC) with about 15% of the cases (Komuta 2021). Although iCCC is relatively rare, its incidence is rising in Western countries (Saha et al. 2016) as does HCC. Early-stage HCC and iCCC often benefit from local ablative therapies such as radiofrequency ablation (RFA) or brachytherapy (EASL 2018).

For all primary liver tumors, targeted therapies (e.g. thymidine kinase 1 and immune checkpoint inhibition) are becoming increasingly important alongside conventional chemotherapy to achieve recurrences rate as low as possible (Finn et al. 2020). In order to allocate the most suitable therapeutic option to each patient, it is of great relevance to have reliable biomarkers allowing for response prediction (Sung et al. 2022; Zhu et al. 2022). Several biomarkers have been analyzed for locoregional therapies that could be indicative for therapy response (Tampaki et al. 2015). Predictive markers include DNA mutations, DNA repair proteins as well as peripheral blood proteins (e.g. AFP, VEGF) and peripheral blood cells (e.g. T lymphocytes) (Ayaru et al. 2007; Eckers and Kimple 2016; Molina et al. 2016). Local ablative treatments not only cause an immediate inflammatory reaction as response to tissue injury, but they can similarly activate certain immune pathways, both enforcing the response to ablation as well as causing synergistic effects with checkpoint inhibition. It has been previously shown that especially low-dose radiation is associated with specific anti-tumorigenic effects within the tumor microenvironment (TME) including macrophage reprogramming and T cell recruitment (Klug et al. 2013; Arnold et al. 2018). As neutrophils seem to be further key players in the progression of liver cancers, therapies targeting neutrophils are recently under investigation (Margetts et al. 2018; Windt et al. 2018). Analyzing the effect of radiation on the TME as well as on systemic immune responses is of major importance in the search for predictive and prognostic biomarkers (Formenti and Demaria 2009; Byrne et al. 2021). As local radiation has specific impact on the cellular composition of tumors, changes of cellular markers of immune activation can potentially serve as markers for response prediction.

Accordingly, we aimed to investigate the potential predictive nature of response of peripheral blood cells in patients with primary liver cancer following interstitial brachytherapy (IBT). Therefore, we analyzed peripheral blood cells of 20 patients and identified a composite signature of thrombocytes and lymphocyte subsets that was linked to patient response.

## Materials and methods

### Patient selection and study design

Patients were recruited in two prospective clinical trials investigating image-guided local ablation of low- and intermediate-stage primary liver cancers. The analysis consists of 20 patients with HCC and iCCC. 15 patients were recruited from the ESTIMATE trial, 5 patients were included from the THIAMAT trial. Peripheral blood samples were obtained at baseline on the day before local ablation as well as up to 72 h post-IBT. Response to treatment was evaluated at 6 months post IBT (Ricke and Wust 2011). An overview of the patients’ clinical characteristics at baseline is shown in Table 1. Analysis of histopathological specimens was performed by clinical pathologists at the Institute of Pathology of the Ludwig-Maximilians University, Germany within the clinical routine.

**Table 1:** Baseline characteristics of the study population pre-IBT.

Patient features	pre-IBT R n=11	pre-IBT NR n=9	p value
Sex			
Female	4	2	0.6424^a^
Male	7	7	
Age at therapy start	70 (±9.12)	72 (±11.37)	0.6626*
Fibrosis	1/11 (5%)	5/9 (56%)	0.0498^a^
Child pugh score			
A	9/11 (82%)	3/9 (33%)	0.0114^a^
B	2/11 (18%)	1/9 (11%)	
NASH	1/11 (9%)	1/9 (11%)	1.0000^a^
High alcohol intake	5/11 (45%)	1/9 (11%)	0.1571^a^
Viral Hepatitis	2/11 18%)	0	0.4789^a^
Average amount of tumors	1.4	1.4	1.0000^a^
Average tumor diameter (all tumors) [mm]	3.20 (2.58)	3.10 (3.30)	0.4931^#^
BCLC			
BCLC A	11/11 (100%)	2/9 (22%)	0.0005^a^
BCLC B	0	2/9 (22%)	
Platelets [G/L]	147.36 (±54.76)	239.22 (±99.06)	0.0169^*^
AFP [ng/mL]	16.65 (250.30)	3.75 (1.50)	0.1071^#^
CA19-9 [U/mL]	n.a.	217.20 (208.81)	
Serum albumin [g/dL]	3.83 (±0.65)	3.91 (±0.41)	0.7407^*^
Total bilirubin [mg/dL]	0.75 (0.90)	0.60 (0.40)	0.4343^#^
ALBI grade			
Grade > 1	4 /11 (36%)	4/9 (44%)	>0.9^a^
Grade 1	6/11 (55%)	5/9 (56%)	
Unknown	1/11 (9%)	0	

The size of tumor lesions was measured as average tumor diameter of all lesion. *AFP* a-fetoprotein, *BCLC* Barcelona Clinic Liver Cancer staging system, *ALBI* Albumin-Bilirubin; *na* not available, *NASH* non-alcoholic steatohepatitis, *NR* non-responder, *R* responder. median (IQR), mean (± SD), *a* Fisher's exact-test, *t-test, # Mann–Whitney U-test.

### Ethics

The clinical study protocol as including biomarker sampling were approved by the local ethics committee of the university hospital (LMU München, Munich, Germany) and listed at the German clinical trial register (ESTIMATE: DRKS 00010587, THIAMAT: DRKS 00010560). All investigations were conducted in accordance with the Declaration of Helsinki. Written informed consent of each participant was obtained prior to enrollment.

### Patient response assessment

Patients were stratified into responders (R) versus non-responders (NR) based on previously published criteria (Kimm et al. 2022) and eligibility for curative versus palliative treatments in case of progression. Accordingly, responders were defined as patients showing complete remission within 6 months following therapy. Any recurrence seen within 6 months post-therapy or tumor appearance in between a total follow-up period of 24 months greater than 3 cm or > 3 tumor lesions classified the patient as non-responder.

### Peripheral blood cell ratios

Lymphocyte-to-monocyte ratio (LMR), neutrophil-to-monocyte ratio (NMR), neutrophil-to-lymphocyte ratio (NLR) and platelet-to-lymphocyte ratio (PLR) were computed by dividing absolute numbers of lymphocytes, monocytes, neutrophils and platelets by the indicated leukocyte population.

### PBMC collection and flow cytometry analysis

**Fig. 1:**
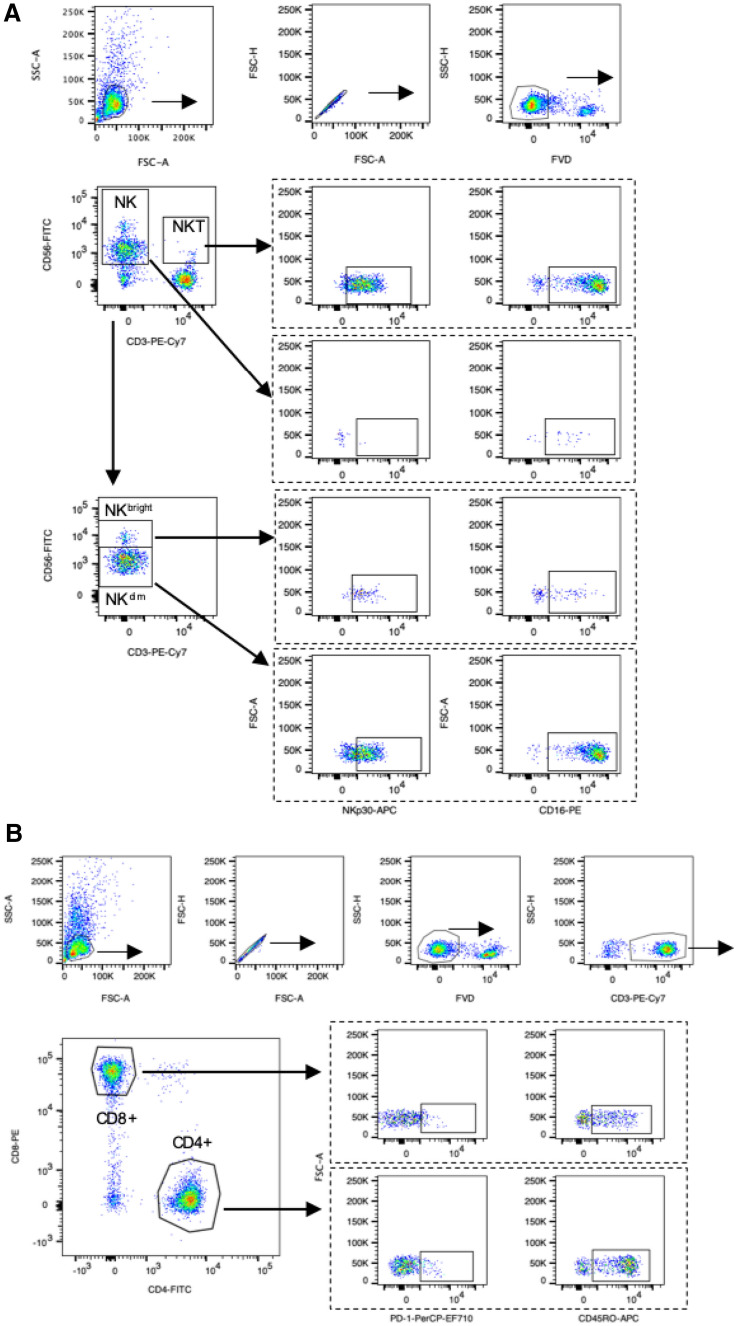
Gating of different lymphoid cell populations. Representative dot plots from one patient. **A** Panel 1: NKT cells were defined as CD3 + CD56 + population (% of viable cells) and subsets as CD3 + CD56 + NKp30 + and CD3 + CD56 + CD16 + cells (% of NKT). NK cells were defined as CD3-CD56 + cells (% of viable cells) and further separated into CD56^bright^ NK and CD56^dim^ NK cells (% of NK cells). NK, CD56^bright^ NK and CD56^dim^ NK cells were further gated for NKp30 + and CD16 + respectively (% of parental population). **B** Panel 2: T cells were defined CD3 + CD4 + or CD3 + CD8 + cells. T cell subsets as CD4 + PD-1 + , CD4 + CD45RO + , CD8 + PD-1 + and CD8 + CD45RO + . All cells are presented as % frequency of viable cells (CD3 +) or the parental population (all T cell subsets). FVD: Fixable Viability Dye-eFluor^®^780

PBMCs from pre- and post- IBT time points were isolated from buffy coats using a density gradient centrifugation protocol and cryopreserved until final analysis. Briefly, whole blood was collected in EDTA-coated tubes and plasma removed after centrifugation (3000 rpm, 10 min, 4 °C). Remaining buffy coat was diluted with an equal volume of 1 × PBS, laid on top of Ficoll-Paque (Cytiva, Uppsala, Sweden) and centrifuged without brake (400 g, 24 min). PBMC were collected from the interphase and frozen in DMSO after red blood cell lysis in ACK buffer (Lonza Ltd, Basel, Switzerland). At day of analysis, cells were thawed at 37 °C and resuspended in staining buffer (1 × PBS/3% FBS). Staining was performed for 30 min at 4 °C in the dark. The following monoclonal antibodies specific for human antigens were used: anti-CD3-PE-Cy7 (OKT3), anti-CD4-FITC (OKT4), anti-CD8-PE (RPA-T8), anti-CD16-PE (B73.1), anti-CD45RO-APC (UCHL1), anti-CD56-FITC (5.1H11) and anti-CD337 (NKp30)-APC (P30-15) (all from BioLegend, San Diego, CA, USA) and anti-PD-1-PerCP-eFluor^®^710 (J105) and Fixable Viability Dye-eFluor^®^780 (all from ThermoFisher Scientific, Waltham, MA, USA). Cells were analyzed on the flow cytometer FACSCanto (BD Biosciences, Immune Cytometry Systems, San Jose, CA, USA), and data were analyzed using FlowJo software version 10 (BD Life Sciences, Ashland, OR, USA). The gates were set based on Fluorescence-minus-one (FMO) and IgG control antibody staining and cells presented as percentage of a defined population. For the calculation of CD4/8 ratios counts of CD4 + and CD8 + T cell populations were used. Gating strategy for NK and CD3 + CD56 + NKT cells (panel 1) is shown in Fig. 1A, gating strategy for T cells (panel 2) is shown in Fig. 1B. CD3 + cells were presented as ratio of viable cells, CD4 + and CD8 + T cell were presented as percentage of CD3 + cells. CD4 + CDPD-1 + and CD4 + CD45RO + were presented as percentage of CD4 + T cells and CD8 + PD-1 + and CD8 + CD45RO + were presented as percentage of CD8 + T cells. CD3-CD56 + NK and CD3 + CD56 + NKT cells were presented as percentage of viable cells, CD56^bright^ NK and CD56^dim^ NK cells as percentage of CD3-CD56 + NK cells. Subsets (NKp30 + and CD16 + cells) were presented as percentage of the respective parental population.

### Statistical analysis

Statistical analysis was performed using GraphPad Prism (version 9, GraphPad Software, San Diego, CA, USA) and SAS (version 9.4, SAS Institute Inc., Cary, NC, USA). Normality distribution was determined by the Shapiro–Wilk-test. Paired data was analyzed using paired *t* test or Wilcoxon test, unpaired data was analyzed using unpaired t-test or Mann–Whitney U-test. For analysis of clinical and demographic data Fisher's exact-test, Mann–Whitney *U* test and *t* test were used in dependency of the normal distribution. Not normally distributed data are presented as median with interquartile range (IQR), normally distributed data as mean with standard deviation (± SD). A *p* value < 0.05 was considered to be significant.

## Results

### Demographic characteristics of the study cohort

Recruitment of all patients took place through the liver clinics in a tertiary care/liver transplant center. Diagnosis of primary liver cancer (HCC, iCCC) was based on radiological criteria and biopsy and made by experienced radiologists and pathologists. Patient characteristics and liver function tests at baseline (pre-IBT) are summarized in Table 1. Already at baseline, patients that did not respond well to IBT showed significant higher platelet counts as patients that responded well (R: 147 G/L, NR: 239 G/L, *p = *0.0169). Serum levels of albumin and bilirubin as well as the resulting ALBI grade did not show noticeable differences. However, differences in fibrosis, Child Pugh score and BCLC score were noted in patients that did not respond well to therapy.

### Cell counts and ratios serve as response prediction markers

**Fig. 2:**
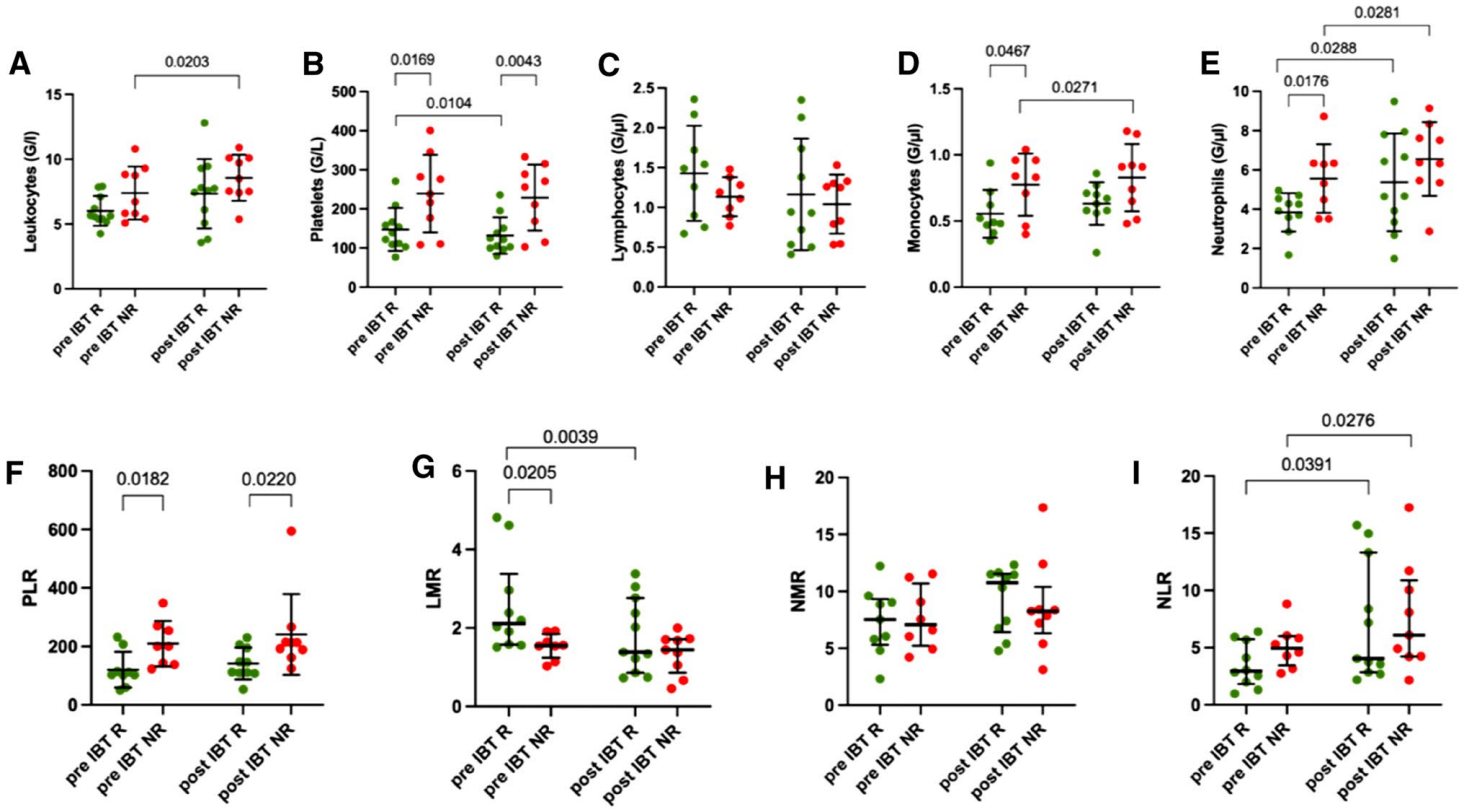
Leukocytes and leukocyte ratios. Pre- and post-IBT analysis of absolute numbers of **A** leukocytes, **B** platelets, **C** lymphocytes, **D** monocytes and **E** neutrophils and ratios **F** PLR, **G** LMR, **H** NMR and **I** NLR. Peripheral blood from 11 therapy-responsive (R, green dots) and 9 therapy non-responsive (NR, red dots) patients were analyzed. Each dot represents an individual patient. Data was analyzed using paired t-test (R: A-F. NR: A-E, G-I), Wilcoxon-test (R: G-I. NR: F), unpaired t-test or (R: A-F, H-I. NR: A-E, G) or Mann–Whitney *U* test (R: G. NR: F, H-I)

As a next step, we analyzed peripheral blood cell counts and ratios of all patients pre- and post-IBT (Fig. 2, Supplementary Table 1). Pre-therapy leukocytes had a tendency to be lower in responders than non-responders (Fig. 2A, Supplementary Table 1). Looking at absolute platelet numbers, responders exhibited lower numbers than non-responders both pre- as well as post-IBT (Fig. 2B, Supplementary Table 1). The absolute numbers of lymphocytes were not indicative for the assessment of therapy response regardless of the time point (Fig. 2C). However, monocytes and neutrophil counts differed significantly between responders and non-responders pre-IBT (Fig. 2D, E), but not post-IBT. For both cell types, responders had lower absolute numbers before brachytherapy (monocytes: 0.56 vs 0.78, *p = *0.0467, neutrophils: 3.85 vs 5.56, *p = *0.0176) (Supplementary Table 1). Furthermore, non-responding patients showed significant increases in absolute monocyte and neutrophil numbers following IBT (Fig. 2 D, E. Supplementary Table 1) whereas responders revealed increased neutrophil counts following IBT (Fig. 2E, Supplementary Table 1).

As various blood cell ratios have been identified as relevant clinical response and prediction markers, we further investigated their potential as response marker following local ablative brachytherapy. PLR was significant lower in responding patients pre- and post-IBT (pre-IBT: 120 vs 210, *p = *0.0182, post-IBT: 142 vs 213, *p = *0.0220). Interestingly, post-IBT ratios did not vary substantially from pre-IBT ratios (Fig. 2F, Supplementary Table 1). Pre-IBT, LMR was significantly higher in responders than non-responders (2.11 vs 1.54, *p = *0.0205), but no differences were found post therapy. Patients that responded well to IBT also showed decreased LMR following therapy (Fig. 2G, Supplementary Table 1). NMR revealed no differences, either in terms of timing or in terms of therapy response (7.36 vs 7.65, *p = *0.8381) (Fig. 2H, Supplementary Table 1). With regard to the time course, NLR increased significantly from pre to post-IBT time points, no matter the patients' response status (R: 3.48 vs 4.04, *p = *0.0391, NR: 5.09 vs 7.63, *p = *0.0276). Regarding the response status, no differences were noted (Fig. 2I, Supplementary Table 1).

### Lymphocyte populations depend on therapy response

To gain further insight into differences in lymphocyte populations pre and post-IBT, we made use of PBMCs isolated from patient's peripheral blood 24 h before and up to 72 h after local ablative therapy and used flow cytometry for subsequent analysis.

In a first setup, we analyzed the percentage of total NK, CD56^dim^ NK and CD56^bright^ NK cells and investigated the expression of the functional receptors CD16 and NKp30 as activation markers in the different populations. Pre-IBT, only the expression of NKp30 significantly differed between responders and non-responders (95.70 vs 87.30, *p = *0.0465) (Supplementary Figure 1, Supplementary Table 2). Overall, no significant differences were seen within NK cell populations no matter of the response state of the patient or the point of time and no obvious response pattern could be identified.

**Fig. 3:**
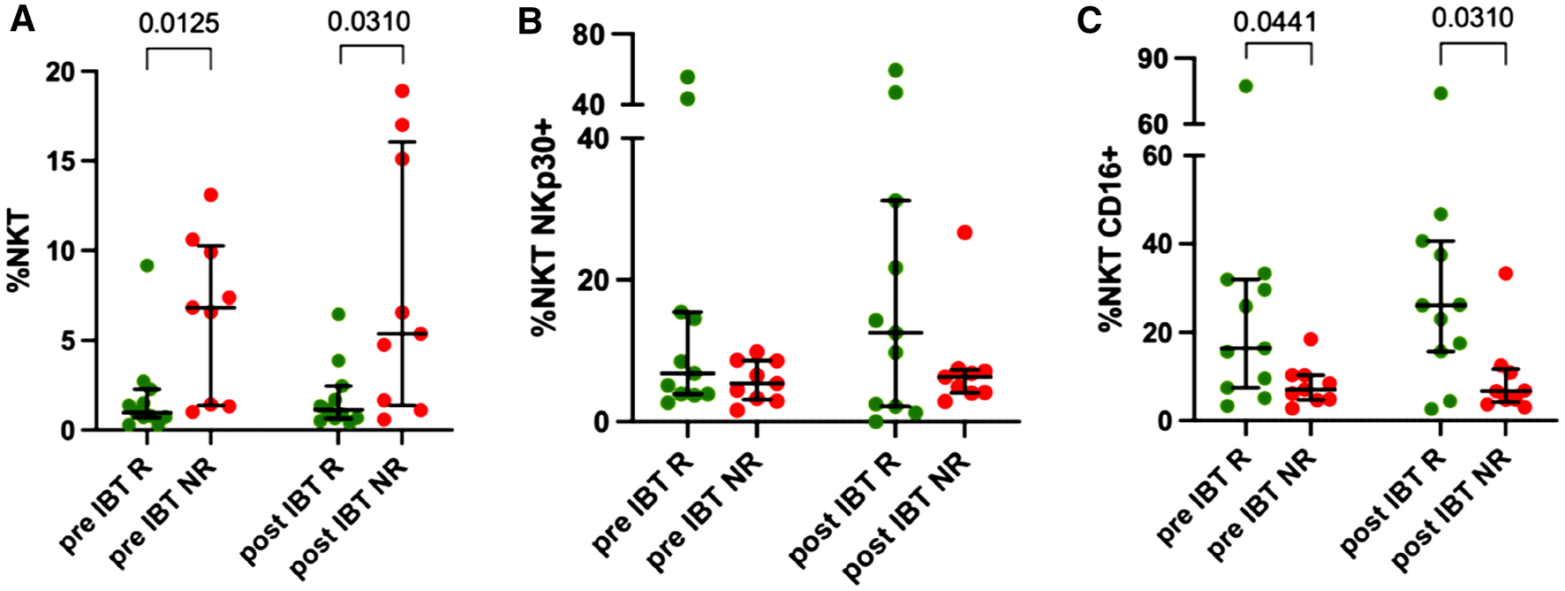
Differences in CD3 + CD56 + NKT cells. Pre- and post-IBT analysis of **A** CD3 + CD56 + NKT cells (% of life), **B** CD3 + CD56 + NKp30 + NKT cells and **C** CD3 + CD56 + CD16 + NKT cells (both % of CD3 + CD56 + NKT cells). R green dots, NR red dots. Each dot represents an individual patient. Data was analyzed using paired t-test (NR: A), Wilcoxon-test (R: A-C. NR: B, C) or Mann–Whitney U-test (R: A-C. NR: A-C)

Next, we analyzed NKT cells as they serve as a bridging population between innate and adoptive immunity. We investigated the percentage of CD3 + CD56 + NKT cells pre- and post-IBT and analyzed the cytotoxicity receptors NKp30 and CD16 within the NKT cell population. Again, we could not detect differences following brachytherapy in the two groups (responder vs non-responder). However, patients that responded well to IBT had significant lower levels of CD3 + CD56 + NKT cells at both time points analyzed compared to non-responding patients (pre-IBT: R: 0.98 vs NR: 6.46, *p = *0.0125, post-IBT: R: 1.13 vs NR: 7.89, *p = *0.0310) (Fig. 3A, Supplementary Table 2). When analyzing NKp30 and CD16 expression, we observed no differences with regard to NKp30 (Fig. 3B, Supplementary Table 2), but observed that non-responding patients had significant lower levels of CD16 receptor on CD3 + CD56 + NKT cells than responder (pre-IBT: R: 16.40 vs NR: 8.11, *p = *0.0441, post-IBT: R: 28.60 vs NR: 6.70, *p = *0.0310) (Fig. 3C, Supplementary Table 2) indicating functional differences in this cell population.

**Fig. 4:**
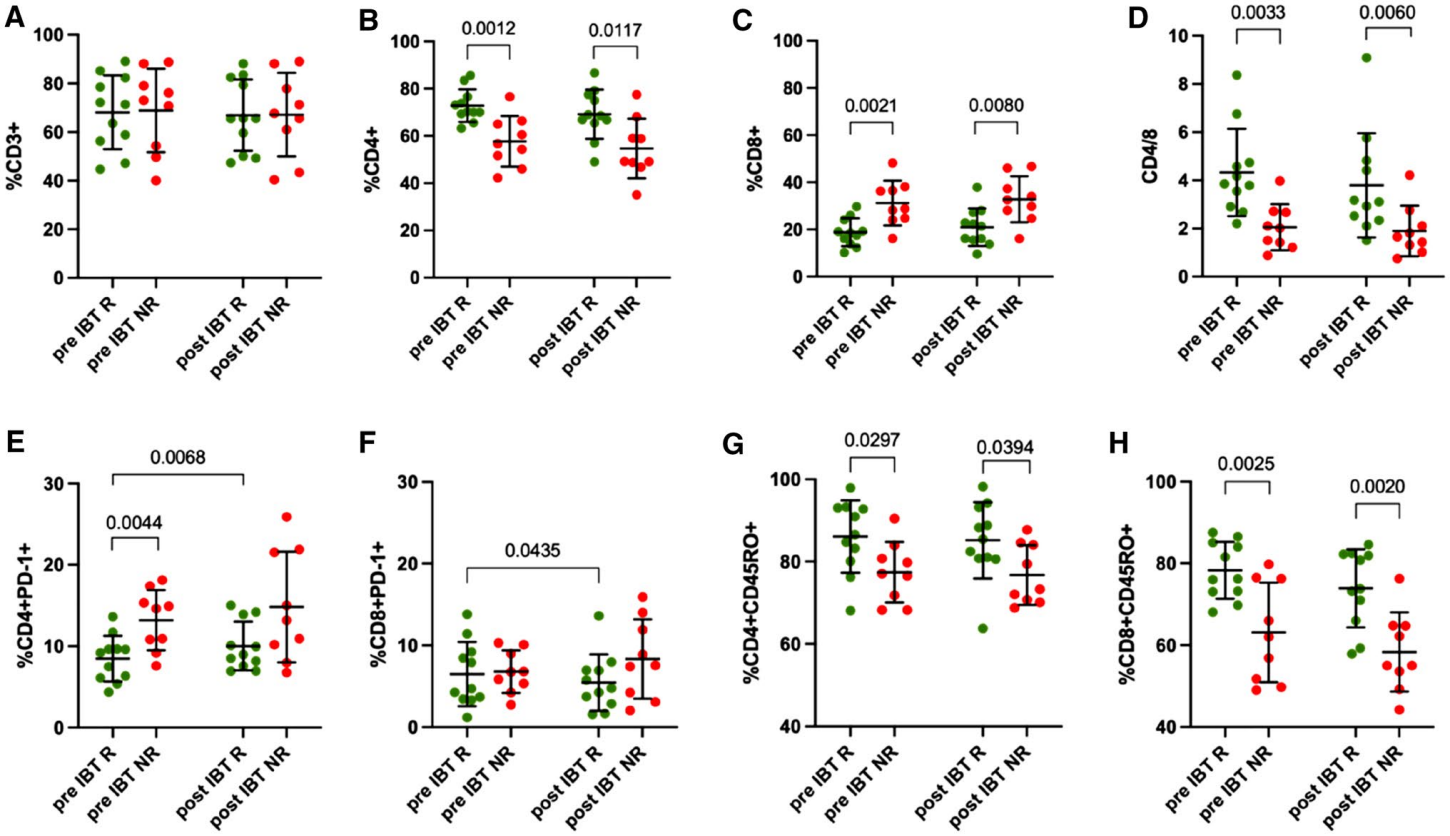
T cell profiling. Pre- and post-IBT analysis of **A** CD3 + T cells (% of life), **B** CD3 + CD4 + T cells (% of CD3 +), **C** CD3 + CD8 + T cells (% of CD3 +), **D** CD4/8 ratio (absolute counts), **E** CD3 + CD4 + PD-1 + T cells (% of CD4 +), CD3 + CD8 + PD-1 + T cells (% of CD8 +), **G** CD3 + CD4 + CD45RO + T cells (% of CD4 +) and **H** CD3 + CD8 + CD45RO + T cells (% of CD8 +). R green dots, NR red dots. Each dot represents an individual patient. Data was analyzed using paired *t* test (R A-C, F–H. NR: A-H), Wilcoxon-test (R E), unpaired t-test (R A-H. NR A-C, F–H or Mann–Whitney *U* test (NR D, E)

Finally, we further analyzed the adoptive immunity by analyzing T cell populations including PD-1 + inhibitor and CD45RO + memory T cells (Fig. 4, Supplementary Table 2). Again, the percentage of CD3 + lymphocytes did not show any differences with regard to therapy response or time point (Fig. 4A) which is in accordance with our results from absolute lymphocyte number results (Fig. 2C). Looking at percentages of CD4 + and CD8 + T cells, significant differences between responder and non-responder were seen, as also evident in the CD4/8 ratio (Fig. 4B–D). Additionally, already pre-IBT non-responders had lower CD4 + T cell levels than responders (R: 73%, NR: 58%. *p = *0.0012) (Fig. 4B). Within the CD4 + T cell population, ratios of inhibitory CD4 + PD-1 + T cells were higher in non-responding patients than in responders (R: 8%, NR: 13%. *p = *0.0044) (Fig. 4E) whereas CD4 + CD45RO + memory T cells were lower (R: 86%, NR: 77%. *p = *0.0297) (Fig. 4G). Looking at CD8 + T cells, we could not detect differences in the CD8 + PD-1 + population (R: 6%, NR: 7%. *p = *0.8461) (Fig. 4F), but did see significantly lower levels of CD8 + CD45RO + memory T cells in non-responding patients (R: 78%, NR: 63%. *p = *0.0025) (Fig. 4H). These observations did not change within 72 h following therapy.

## Discussion

Biomarkers are not only essential for patient’s diagnosis, but are also indispensable in predicting therapy response and tumor recurrence. Early-stage primary liver cancers for whom surgical resection is not an option often benefit from local ablative therapies. However, more than 50% of these patients experience tumor recurrence within 5 years following ablation (Wang et al. 2012; Cao et al. 2022). Early response prediction reflecting the behavior of both the tumor and TME as well as the immune system could help to identify non-responding patients which could then promptly be allocated to further therapy. Traditional tissue biopsies reflect the cellular composition of tumor and TME whereas the analysis of peripheral blood may allow the drawing of conclusions about the systemic impact on the immune system and activation of innate and adaptive immune processes. Liquid biopsies as non-invasive technology provides the possibility to analyze for instance circulating tumor DNA and extracellular vesicles (Alunni-Fabbroni et al. 2019; Felden et al. 2020; Shuen et al. 2022) and recent data clearly shows a substantial impact of liquid biopsies in the field of biomarker research (Maravelia et al. 2021). Furthermore, analysis of peripheral blood cells may not only be suitable for general screening of primary liver cancers and therapy response, but may help to gain insight in the functional state of cellular immune responses. Of special interest are immune cells, as irradiation of tumor cells can induce both, immunosuppressive and immunostimulatory effects (Fleischmann et al. 2021). On the one hand, DNA damage in tumor cells due to ionizing radiation leads to an increase in the overall mutational load. On the other hand, novel acquired mutations in tumor cells, caused by irradiation, can function as tumor neoantigens which can be of strong immunogenicity, thereby causing and amplifying cancer immune-surveillance (DuPage et al. 2013; Gubin et al. 2015; Alspach et al. 2019). Recently, the concept of synergistic radiation therapy and immunotherapy was strengthened by showing that tumor cells that were treated with noncurative doses of irradiation induced somatic mutations that were successfully targeted by anti-PD-1/anti-CTLA-4 immunotherapy (Lussier et al. 2021). Once more, biomarkers identifying immunogenic changes that allow the identification of therapy responsiveness of patients pre-therapy represent an urgent clinical need.

Our study reveals the extent of immune alteration after local therapy of primary liver cancer, with a specific immune cell signature being associated with early tumor recurrence.

Leukocytosis is often linked to tumor recurrence (Schernberg et al. 2018; Zhang et al. 2020) and our results are concordant as non-responders showed increased leukocyte counts at baseline. Furthermore, platelet counts and PLR were increased in non-responders pre- and post- therapy which can hint towards a decrease in liver function and may correspond with tumor burden. In general, liver tumors develop in a pre-damaged organ in which chronic inflammation provides optimal conditions for establishing a supportive tumor microenvironment. Due to leaky blood vessels tumor cells can lead to the activation of the coagulation cascade (Pavlovic et al. 2019). Recently, platelet RNA has been reported as biomarker to differentiate between late-stage cirrhotic nodules and early-stage HCC (Waqar et al. 2021). In addition, platelet counts have been shown to correlate with survival of HCC patients (Lu et al. 2020) and in a preclinical model where antiplatelet therapy improved the survival of HCC bearing mice (Sitia et al. 2012).

Further, we detected lower baseline numbers of monocytes and neutrophils in responders which in turn could indicate a decrease in hepatic inflammation (Shen et al. 2014; Mao et al. 2015). Moreover, we found a strong correlation of LMR with therapy response at baseline where responders had a higher LMR.

Lymphocytes can be tumor promoting or tumor suppressive and with respect to CD56 + NK cells, non-responders of our cohort showed lower levels of NKp30 + NK cells than responders. This could reflect an immune escape mechanism by the tumor cells as the cytotoxic behavior of NK cells is limited. Immunosuppressive isoforms of NKp30 have been described in gastrointestinal tumors and liver cancer and their appearance correlated with a worse prognosis (Delahaye et al. 2011; Mantovani et al. 2019). Whether NKp30 was downregulated in our patient cohort or whether different isoforms are present in the CD56 + NK cells requires further investigation. NKT cells are a relatively small subset of lymphocytes bridging innate and adaptive immunity (Kaer et al. 2011). Compared to responders, we found higher levels of circulating NKT cells, but lower levels of CD16 + NKT cells in non-responders. As CD16 is involved in cell-mediated cytotoxicity (Mandelboim et al. 1999; Yeap et al. 2016; Krijgsman et al. 2018) its downregulation of it might further indicate how liver tumors escape the immune response. Only recently, dysfunctional NKT cells were found in HCC patients.

Furthermore, it has been demonstrated that anti-PD-1 blockade was able to rescue these dysfunctional NKT cells (Tao et al. 2021). This is of particular interest as our study also revealed comparable higher ratio of CD4 + PD-1 + T cells in non-responders, suggesting that these patients could benefit from additional anti-PD-1 checkpoint inhibitory therapy. Further studies are needed to analyze if patients with liver cancer also harbor circulating PD-1 + NKT cells which could be used as biomarker as has been shown for melanoma patients (Bochem et al. 2019). Although we found an increase in CD4 + PD-1 + T cells in non-responders pre-therapy, no changes or differences were observed in CD8 + PD-1 + T cell population.

When analyzing the total CD4 + and CD8 + T cell population, we found comparatively few CD4 + T cells and high levels of CD8 + T cells in non-responders. Interestingly, CD4 + T cell levels in responders reflect the situation in healthy donors. Non-responders however showed lower levels in CD4 + T cell population, but increased percentage of CD4 + PD-1 + T cells within the CD4 + T cell population. When looking into details if there are differences in the memory subset of CD4 + and CD8 + T cells, we found significant changes between responders and non-responders. Responders showed higher levels of CD4 + CD45RO + and CD8 + CD45RO + T cells which could point to a more protective immune state in the therapy-responsive patients. Decreases in the quantity of CD4 + tumor infiltrating T cells (TIL) have been described for advanced-stage HCC suggesting a reduction could indicate tumor progression (Fu et al. 2013). The possible relation between increasing numbers of circulating CD4 + PD-1 + T cells in non-responders and the described CD4 + TIL reduction needs further investigations. As CD4 + CD45RO + TILs were already correlated with increased survival in patients with gastric cancer (Lee et al. 2008; Pagès et al. 2009), we propose that the higher ratios of circulating CD4 + CD45RO + and CD8 + CD45RO + T cells in peripheral blood of patients with primary liver cancers may serve as useful biomarkers to predict response to brachytherapy.

From a developmental point of view, HCC and iCCC share common genomic characteristics (Hoadley et al. 2018) and are grouped as primary liver cancers. Nevertheless, both entities also harbor many differences that makes it indispensable for treatment and prediction to look into more detail into both types of tumors. As iCCC is a relatively rare disease, we grouped all primary liver tumor patients treated with brachytherapy together in order to identify first common markers. This resulted in the fact that the non-responder group contained a higher proportion of iCCC compared to HCC, which is a clear limitation of our study. Additionally, overall, the sample size of this study was small allowing only a limited amount of subgroup analysis. A third limitation is the limited kinetic study of immune cells after local ablation, as only one time point after brachytherapy was investigated. Distinct immune cell populations might serve as predictive biomarkers when observed at later points after brachytherapy. This will be remedied in future studies.

In conclusion, we observe that several peripheral blood-based biomarkers predict the response to interstitial brachytherapy, potentially reflecting alterations of the tumor microenvironment as well as the systemic immune response towards local ablation. This signature consisting of platelet, monocytes, and dedicated neutrophil and lymphocyte subsets, allowing to distinguish between responders and non-responding patients with primary liver cancer.

## Supplementary Information

Below is the link to the electronic supplementary material.

Supplementary file1 (DOCX 720 KB)

## Data Availability

The datasets generated during and/or analyzed during the current study are available from the corresponding author on reasonable request.
