# Supplementary material for: Peripheral blood-based cell signature indicates response to interstitial brachytherapy in primary liver cancer
Source: J Cancer Res Clin Oncol. 2023 May 29;149(12):9777–86. doi: 10.1007/s00432-023-04875-z (PMC10423129; doi:10.1007/s00432-023-04875-z)
Supplement: Supplementary file 1 — Supplementary file1 (DOCX 720 KB) [file 432_2023_4875_MOESM1_ESM.docx]

**Peripheral blood-based cell signature indicates response to interstitial brachytherapy in primary liver cancer**

Sophia Kästle^1,#^, Matthias R. Stechele^1,#^, Lisa Richter^2^, Regina Schinner^1^, Elif Öcal^1^, Marianna Alunni-Fabbroni^1^, Enrico De Toni^3^, Stefanie Corradini^4^, Max Seidensticker^1^, S. Nahum Goldberg^5,6,7^, Jens Ricke^1^, Moritz Wildgruber^1^, Melanie A. Kimm^1*^

^1^ Department of Radiology, University Hospital, LMU Munich, Munich, Germany

^2^ Core Facility Flow Cytometry, Biomedical Center Munich, Ludwig-Maximilians-Universität München, Planegg-Martinsried, Germany

^3^ Department of Medicine II, University Hospital, LMU Munich, Munich, Germany

^4^ Department of Radiation Oncology, University Hospital, LMU Munich, Munich, Germany

^5^ Goldyne Savad Institute of Gene Therapy, Hadassah Hebrew University Hospital, Jerusalem, Israel

^6^ Laboratory for Minimally Invasive Tumor Therapies, Department of Radiology, Beth Israel Deaconess Medical Center, Harvard Medical School, Boston, MA, USA

^7^ Division of Image-guided Therapy and Interventional Oncology, Department of Radiology, Hadassah Hebrew University Hospital, Jerusalem, Israel

* Corresponding author

# equal contribution

**Correspondance to**:

Melanie A. Kimm,

Department of Radiology,

University Hospital LMU Munich,

Marchioninistraße 15,

81377 Munich, Germany

phone ++49-89-4400 73250

melanie.kimm@med.uni-muenchen.de

Orcid: 0000-0001-6833-9738

**Supplementary Information**

**
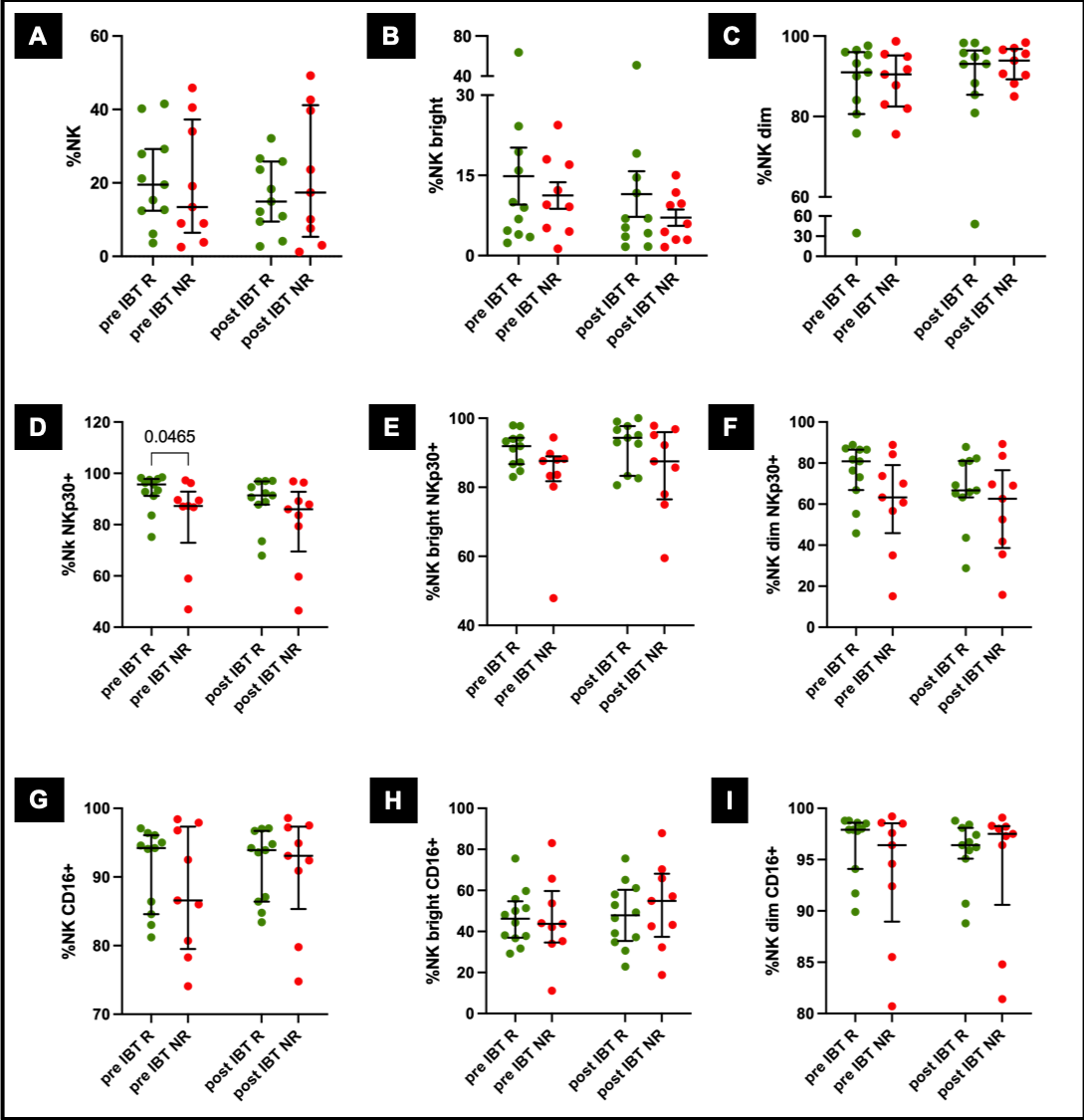
**

**Supplementary Figure 1. Characterization of peripheral NK cells pre- and post-IBT.** Pre and post-IBT analysis of (A) NK (% of viable cells), (B) CD56^bright^ NK (% of NK), (C) CD56^dim^ NK (% of NK), (D) NKp30+NK (% of NK), (E) NKp30+CD56^bright^ NK (% of CD56^bright^ NK), (F) NKp30+CD56^dim^ NK (% of CD56^dim^ NK), (G) CD16+NK (% of NK), (H) CD16+CD56^brigh^ NK (% of CD56^bright^ NK) and (I) CD16+CD56^dim^ NK cells (% of CD56^dim^ NK). Analysis of 11 therapy-responsive (R, green dots) and 9 therapy non-responsive (NR, red dots) patients. Each dot represents an individual patient. Data was analyzed using paired t-test (R: A, E, H. NR: A-C, F, H), Wilcoxon-test (R: B-D, F, G, I. NR: D, E, G, I), unpaired t-test or (R: A, H. NR: A, F, H) or Mann-Whitney U-test (R: B-G, I. NR: B-E, G, I). A *p* value < 0.05 indicates statistical significance.

**Supplementary Table 1. Numerical overview of leukocytes and ratios.**

|  | **pre IBT R** | **post IBT R** | ***p (pre vs post R)*** | **pre IBT NR** | **post IBT NR** | ***p (pre vs post NR)*** | ***p (pre R vs NR)*** | ***p (post R vs NR)*** |
| --- | --- | --- | --- | --- | --- | --- | --- | --- |
| **Leukocytes [G/µL]** | 6.00 (±1.15) | 7.46 (±2.65) | *0.0915^a^* | 7.00 (±1.72) | 8.07 (±1.55) | *0.0203^a^* | *0.0731^c^* | *0.2526^c^* |
| **Platelets [G/µL]** | 147.36 (±54.76) | 132 (±46.75) | *0.0140^a^* | 239.22 (±99.06) | 229.11 (±84.48) | *0.5148^a^* | *0.0169^c^* | *0.0043^c^* |
| **Lymphocytes [G/µL]** | 1.43 (±0.56) | 1.16 (±0.67) | *0.1711^a^* | 1.14 (±0.23) | 1.04 (±0.35) | *0.1739^a^* | *0.2158^c^* | *0.6448^c^* |
| **Monocytes [G/µL]** | 0.56 (±0.17) | 0.63 (±0.15) | *0.1458^a^* | 0.78 (±0.22) | 0.82 (±0.24) | *0.0271^a^* | *0.0467^c^* | *0.0572^c^* |
| **Neutrophils [G/µL]** | 3.85 (±0.93) | 5.37 (±2.37) | *0.0288^a^* | 5.56 (±1.63) | 6.55 (±1.77) | *0.0281^a^* | *0.0176^c^* | *0.2552^c^* |
| **PLR** | 120.42 (±60.98) | 141.70 (±54.51) | *0.3591^a^* | 209.58 (±77.66) | 212.96 (26.19) | *0.3125^b^* | *0.0182^c^* | *0.0220^d^* |
| **LMR** | 2.11 (1.15) | 1.81 (±0.91) | *0.0039^b^* | 1.54 (±0.30) | 1.35 (±0.49) | *0.0562^a^* | *0.0205^d^* | *0.2117^c^* |
| **NMR** | 7.36 (±2.78) | 10.76 (4.57) | *0.8438^b^* | 7.65 (±2.57) | 8.71 (±3.86) | *0.2216^a^* | *0.8381^c^* | *0.7197^d^* |
| **NLR** | 3.48 (±1.85) | 4.04 (7.64) | *0.0391^b^* | 5.09 (±1.78) | 7.63 (±4.44) | *0.0276^a^* | *0.0984^c^* | *0.5516^d^* |

NR: non-responder; R: responder. median (IQR), mean (±SD). a: paired t-test, b: Wilcoxon test, c: unpaired t-test, d: Mann-Whitney U-test. A *p* value < 0.05 indicates statistical significance.

**Supplementary Table 2. Numerical overview over lymphocytes.**

|  | **pre IBT R** | **post IBT R** | ***p (pre vs post R)*** | **pre IBT NR** | **post IBT NR** | ***p (pre vs post NR)*** | ***p (pre R vs R)*** | ***p (post R vs NR)*** |
| --- | --- | --- | --- | --- | --- | --- | --- | --- |
| **CD3+** | 68.14 (±14.47) | 66.97 (±14) | *0.6877^a^* | 68.86 (±16.18) | 67.16 (±16.23) | *0.4605^a^* | *0.9219^c^* | *0.9798^c^* |
| **CD4+** | 72.85 (±6.55) | 69.20 (±9.96) | *0.0784^a^* | 57.73 (±10.09) | 54.72 (±11.95) | *0.1916^a^* | *0.0012^c^* | *0.0117^c^* |
| **CD8+** | 18.80 (±5.62) | 20.98 (±7.62) | *0.1104^a^* | 31.23 (±8.96) | 32.80 (±9.22) | *0.2563^a^* | *0.0021^c^* | *0.0080^c^* |
| **CD4/8** | 4.33 (±1.73) | 3.10 (2.19) | *0.0830^b^* | 2.05 (±0.90) | 1.89 (±0.99) | *0.2433^a^* | *0.0033^c^* | *0.0060^d^* |
| **CD4+PD-1+** | 8.46 (±2.68) | 10.02 (2.85) | *0.0068^b^* | 13.19 (±3.50) | 14.81 (±6.40) | *0.3810^a^* | *0.0044^c^* | *0.1162^d^* |
| **CD8+PD-1+** | 6.49 (±3.75) | 5.46 (±3.28) | *0.0435^a^* | 6.79 (±2.45) | 8.34 (±4.57) | *0.2175^a^* | *0.8461^c^* | *0.1384^c^* |
| **CD4+CD45RO+** | 86.07 (±8.40) | 85.15 (±8.84) | *0.4122^a^* | 77.39 (±6.91) | 76.73 (±6.83) | *0.7125^a^* | *0.0297^c^* | *0.0394^c^* |
| **CD8+CD45RO+** | 78.28 (±6.61) | 73.92 (±9.08) | *0.1347^a^* | 63.12 (±11.47) | 58.33 (±9.12) | *0.1678^a^* | *0.0025^c^* | *0.0020^c^* |
| **NKT** | 0.98 (1.18) | 1.13 (1.42) | *0.7646^b^* | 6.46 (±4.16) | 7,89 (±6.77) | *0.2371^a^* | *0.0125^d^* | *0.0310^d^* |
| **NKT NKp30+** | 6.84 (11.14) | 12.6 (24.12) | *0.5771^b^* | 5.72 (±2.74) | 6.32 (3.03) | *0.7344^b^* | *0.3596^d^* | *0.5027^d^* |
| **NKT CD16+** | 16.40 (22.26) | 28.60 (±19.52) | *0.0830^b^* | 8.11 (±4.39) | 6.70 (6.16) | 0.5469^b^ | *0.0441^d^* | *0.0310^d^* |
| **NK** | 20.88 (±12.08) | 16.43 (±9.19) | *0.1170^a^* | 19.69 (±15.42) | 21.61 (±17.12) | *0.3342^a^* | *0.8569^c^* | *0.4239^c^* |
| **NK bright** | 2.39 (13.33) | 6.92 (9.27) | *0.1748^b^* | 11.24 (±7.01) | 7.10 (±4.34) | *0.0660^a^* | *0.8820^d^* | *0.7103^d^* |
| **NK dim** | 91.00 (13.30) | 93.10 (9.25) | *0.1748^b^* | 88.86 (±7.04) | 92.84 (±4.29) | *0.0709^a^* | *0.9262^d^* | *0.7664^d^* |
| **NK NKp30+** | 95.70 (5.65) | 91.40 (7.40) | *0.0674^b^* | 87.30 (2.80) | 80.63 (±15.92) | *0.2383^b^* | *0.0465^d^* | *0.1354^c^* |
| **NK_bright_ NKp30+** | 91.08 (±4.81) | 92.25 (±6.58) | *0.5221^a^* | 82.54 (4.90) | 85.29 (±11.84) | *0.4258^b^* | *0.0952^d^* | *0.1322^c^* |
| **NK_dim_ NKp30+** | 75.37 (±13.46) | 66.74 (±16.71) | *0.0568^a^* | 60.86 (±22.04) | 57.73 (±22.33) | *0.3383^a^* | *0.1033^c^* | *0.3412^c^* |
| **NK CD16+** | 94.20 (9.75) | 93.90 (9.00) | *0.5410^b^* | 87.92 (±8.50) | 93.10 (6.30) | *0.1602^b^* | *0.6556^d^* | *0.8238^d^* |
| **NK_bright_ CD16+** | 46.53 (±12.60) | 47.75 (±14.86) | *0.7111^a^* | 45.84 (±19.20) | 52.54 (±19.74) | *0.1020^a^* | *0.9268^c^* | *0.5530^c^* |
| **NK_dim_ CD16+** | 97.90 (2.60) | 96.40 (2.25) | *0.0605^b^* | 96.40 (6.10) | 97.50 (1.80) | *0.6797^b^* | *0.4006^d^* | *0.5647^d^* |

NR: non-responder; R: responder. median (IQR), mean (±SD). a: paired t-test, b: Wilcoxon test, c: unpaired t-test, d: Mann-Whitney U-test.
